# Expanding the spectrum of *AFF2* carcinoma: clinical, morphological, immunohistochemical, and molecular characteristics of five cases harboring alternate fusions

**DOI:** 10.1007/s00428-025-04140-3

**Published:** 2025-06-10

**Authors:** Gerben E. Breimer, Martin D. Hyrcza, Elan Hahn, Sophie C. Prendergast, Stephen M. Smith, Anne Chambers, Emma Todorovic, Doreen Palsgrove, Daniel L. Miller, Robert J. Heinhuis, Johannes A. Rijken, Lennart A. Kester, Cuihong Wei, Ilan Weinreb, Justin A. Bishop

**Affiliations:** 1 Department of Pathology, University Medical Center Utrecht, Utrecht, The Netherlands; 2 Department of Pathology and Laboratory Medicine, University of Calgary, Arnie Charbonneau Cancer Institute, Calgary, AB Canada; 3 Department of Pathology and Laboratory Medicine, Sinai Health System, Toronto, ON Canada; 4 Department of Laboratory Medicine and Pathobiology, University of Toronto, Toronto, ON Canada; 5 Laboratory Medicine Program, University Health Network, University of Toronto, Toronto, ON Canada; 6 Department of Pathology, Royal Victoria Infirmary, The Newcastle Upon Tyne Hospitals NHS Foundation Trust, Newcastle Upon Tyne, UK; 7 Department of Pathology and Laboratory Medicine, University of Texas Southwestern Medical Center, Dallas, TX USA; 8 Department of Pathology, Saint Louis University School of Medicine, St Louis, MO USA; 9 PAL, Laboratory for Pathology Dordrecht, Dordrecht, The Netherlands; 10 Department of Head and Neck Surgical Oncology, University Medical Center Utrecht, Utrecht, The Netherlands; 11 Department of Pathology, Princess Máxima Center for Pediatric Oncology, Utrecht, The Netherlands

**Keywords:** Head and neck neoplasms, Gene fusion, Sinonasal neoplasms, Immunohistochemistry, RNA sequencing, DEK::AFF2 fusion

## Abstract

In recent years, multiple molecularly defined entities have emerged in head and neck pathology, especially among sinonasal squamous and basaloid carcinomas, including NUT carcinoma, SWI/SNF-deficient carcinoma, and *DEK*::*AFF2* carcinoma. These tumors show significant morphological and immunophenotypic diversity. We present five novel head and neck carcinomas harboring *AFF2* rearrangements involving previously unreported fusion partners. Five cases (3 males, 2 females; ages 35–72 years) presented with tumors in the sinonasal region (*n* = 4) and parotid gland (*n* = 1), measuring between 3.3 and 6.3 cm. RNA sequencing identified fusions involving *AFF2* with *H3-3A*, *EWSR1*, *CHD4* (two cases: neck lymph node metastasis, which turned out to be sinonasal primary and parotid mass), and *NUCKS1*. Tumors harboring *H3-3A*::*AFF2* and *NUCKS1*::*AFF2* fusions exhibited bland transitional cell-like morphology with acantholytic changes similar to classic *DEK*::*AFF2* carcinoma; the *NUCKS1* fusion also demonstrated clear cell features. In contrast, the *EWSR1*::*AFF2* fusion tumor showed high-grade adenocarcinoma morphology with focal neuroendocrine marker expression, lacking p63 and CK5/6. The two *CHD4*::*AFF2* fusion cases demonstrated neuroendocrine differentiation; one was a cytokeratin-negative small blue round cell carcinoma, and the other showed mixed squamoid-neuroendocrine features with strong cytokeratin and p63 expression. All tumors demonstrated consistent AFF2 immunoreactivity. These findings suggest that *AFF2*-rearranged tumors form a spectrum of carcinomas with diverse morphologies, immunophenotypes, and differentiation patterns. Given the consistent involvement of the *AFF2* gene and uniform AFF2 immunohistochemical positivity despite morphological heterogeneity, we propose naming this entity AFF2 carcinoma.

## Introduction

The widespread availability of molecular RNA and DNA-based analysis has advanced identifying and characterizing specific sinonasal cancer types, such as NUT carcinoma, SWI/SNF deficient carcinoma, and *DEK*::*AFF2* carcinoma. Although these tumors are often initially described based on their unique morphological features, it soon becomes apparent that they can exhibit a diverse range of morphologies and immunophenotypes. For example, NUT carcinoma is typically composed of sheets of monomorphous small blue round cells with areas of abrupt keratinization [1]. However, various other architectural patterns and morphologies have been documented [2, 3].

*DEK*::*AFF2* carcinoma is recognized as a non-keratinizing, transitional-type squamous cell carcinoma, primarily arising in the sinonasal region and skull base, with a characteristic histologic appearance, including neutrophilic infiltration and occasional resemblance to inverted papilloma [4, 5]. However, Miller et al. reported a high-grade neuroendocrine tumor with a *CHD4*::*AFF2* fusion, raising the question of whether neoplasms with *AFF2* alterations represent a broader morphological and immunophenotypic spectrum than previously thought [6].

The *AFF2* gene (AF4/FMR2 family member 2) is located on the X chromosome in the Xq28 locus. It encodes a putative transcriptional factor, whose function is currently not well understood [7, 8]. The AFF2 protein is usually expressed in bone marrow, brain, and placental tissue [8]. It is a subunit of the SEC-like complex SEC-L2, which is required for transcription elongation of specific genes [9]. This complex is known to regulate RNA polymerase II activity by preventing its paused state [10]. However, the precise cellular functions of AFF2 remain elusive. Mutations in the *AFF2* gene are associated with intellectual disability, Cornelia de Lange syndrome, and Fragile X syndrome [11]. *AFF2* fusions have been observed in a subset of squamous cell carcinomas (typically reported with fusion partner *DEK*), and in rare cases of sarcomas (*WWTR1*::*AFF2*), lymphomas (*STAG2*::*AFF2* and *TOP2B*::*AFF2*), and in the central nervous system neuroepithelial tumor (*LARGE1*::*AFF2*) [8, 12–14].

Despite the enlarging spectrum of *AFF2-*fusion neoplasms, there remains a knowledge gap concerning variations of fusion partners and their associated morphologies and immunophenotypic characteristics. To address this, our study presents five cases of head and neck carcinomas with alternative fusion partners, each demonstrating diverse morphologies and immunohistochemical profiles. By examining these cases, we aim to expand the current understanding of the morphological and immunophenotypic spectrum of *AFF2*-fusion-driven tumors and contribute to more precise diagnostic and therapeutic approaches.

## Methods

Five tumors were identified from the authors’ consultation and surgical pathology archives. One of these cases had been previously published [6]. The tissue specimens were formalin-fixed and processed according to standard histopathological protocols. Due to the consultation-based nature of these cases, immunohistochemistry (IHC) was conducted in various laboratories, resulting in variability in the applied stains depending on tissue availability and the initial differential diagnosis. Detailed staining protocols and antibody sources are available upon request.

### Next generation sequencing

For case 1, whole transcriptome RNA sequencing was performed as previously described [15]. RNA sequencing libraries were generated using 300 ng RNA using the KAPA RNA HyperPrep Kit with RiboErase (Roche). Sequencing was performed on a NovaSeq 6000 system (2 × 150 base pairs, Illumina). The resulting reads were aligned using Star fusion (version 2.7.0f) to GRCh38 and gencode version 29.

For case 2, DNA and RNA were extracted from formalin-fixed paraffin-embedded (FFPE) using the Qiagen AllPrep DNA/RNA FFPE tissue kit with the following modifications: crosslinking reversal at 80 degrees Celsius was performed for 1 h, and DNAse was omitted. RNA was converted to cDNA, and library preparation was performed using ArcherDx FusionPlex reagents (Invitae) for Ion Torrent™, with a custom primer design. Next-generation sequencing (NGS) was performed using an Ion Torrent Genexus System (ThermoFisher Scientific), software version 6.6. Sequence output files were analyzed on Archer Analysis v6.2.7 (ArcherDX, Inc.). File transfers and additional data processing were performed using custom in-house Python™ scripts. All results were reviewed by a molecular pathologist.

For case 3, targeted RNA sequencing (RNA-Seq) was performed, as described elsewhere [16]. In brief, RNA was isolated from 10 µm whole-slide tissue sections using Qiagen AllPrep kits (Qiagen, Germantown, MD). A modified TruSight RNA Pan-Cancer kit (Illumina, San Diego, CA) was subsequently used to make a sequencing library containing all exons from 1516 known cancer-related genes. Sequencing was performed on the NextSeq 550 (Illumina), with a minimum of 6,000,000 mapped reads. All fusions and variants were reviewed in the Integrated Genomics Viewer (Broad Institute, Cambridge, MA). The Star-Fusion algorithm was used to call fusions.

For cases 4 and 5, RNA was extracted from the submitted tissue and analyzed using the UHN CST Panel Version 1.0. For fusions, all exons of targeted genes defined in the RefSeq transcript set are examined using the Illumina RNA Prep protocol. The RNA library is enriched by hybrid capture followed by paired-end sequencing with the Illumina sequencing platform. Variant calls are generated using the UHN Clinical Laboratory Genetics custom bioinformatics pipeline with alignment to genome build GRCh37/hg19, and variants are assessed using custom scripts. Fusions are analyzed using STAR-Fusion and CTAT-Splicing [17].

## Results

We report five carcinomas, affecting three males and two females aged 35 to 72 years; see Table 1 for patient characteristics and Table 2 for immunohistochemistry findings. RNA sequencing detected *H3-3A*::*AFF2*, *EWSR1*::*AFF2*, *CHD4*::*AFF2* (2), and *NUCKS1*::*AFF2* fusions; see Table 3 for details on fusion exons and mutations.

**Table 1:** Patient characteristics

No	Age/sex	Site (size in cm)	Original/submitted diagnosis	Histological pattern	TNM	Treatment	Outcome	Molecular analysis
1	52/F	Maxillary sinus (3.3 cm)	Non-keratinizing squamous cell carcinoma	Bland non-keratinizing squamous cell carcinoma with dissociative growth	pT2N0 cM0	Endonasal endoscopic resection with postoperative radiotherapy	12 months follow-up, NED	*H3-3A*::*AFF2*
2	35/M	Nasal cavity (6.3 cm)	High-grade sinonasal non-intestinal adenocarcinoma	High-grade carcinoma with glandular and small round blue cell tumor morphology areas	cT4bN2M0	RT 70 Gy in 33 fractions	17 months follow-up, NED	*EWSR1*::*AFF2*
3	49/M	Lymph node metastasis (3.0 cm)	Metastatic high-grade neuroendocrine tumor of unknown primary	Small round blue cell tumor	pT0N3b cM0	Neck dissection, left side levels I–IV. pT0N3b cM0. Adjuvant radiation and chemotherapy	37-month follow-up, primary left nasolacrimal with multiple right-sided metastases, for which maxillectomy with orbital exenteration and right neck dissection, followed by radiation and chemotherapy	*CHD4*::*AFF2*
4	72/M	Nasal cavity (size N/A)	NA	Bland non-keratinizing squamous cell carcinoma with clear cell changes	N/A	N/A	N/A	*NUCKS1*::*AFF2*
5	70/F	Right parotid gland (4.2 cm)	High-grade carcinoma with neuroendocrine differentiation	Predominantly non-keratinizing squamous cell carcinoma with neuroendocrine features	pT3N1 cM0	Right total parotidectomy with facial nerve preservation; pending adjuvant radiotherapy	2-month follow-up, NED	*CHD4*::*AFF2*

**Table 2:** Immunohistochemistry findings

	Case 1	Case 2	Case 3	Case 4	Case 5
Gene fusion	*H3-3A*::*AFF2*	*EWSR1*::*AFF2*	*CHD4*::*AFF2*	*NUCKS1*::*AFF2*	*CHD4*::*AFF2*
AFF2	+	+	+	+	+
Keratin AE1/AE3	+	+	-	NA	+
p40	+	-	-	NA	+
p63	+	-	Weakly positive	NA	+
Keratin 5	Variable positivity	-	-	NA	+
p16	Patchy	Patchy	Negative	Negative	NA
BRG1	Retained	Retained	Retained	NA	NA
INI1	Retained	Retained	Retained	NA	NA
EBER ISH	-	-	-	-	-
CD99	-	-	+ (F)	NA	NA
SOX10	-	-	-	NA	-
Synaptophysin	-	+ (F)	+	NA	+
Chromogranin A	NA	+ (F)	+	NA	+
Desmin	-	-	-	NA	NA
NUT	-	-	-	NA	NA

**Table 3:** Details on fusion exons and mutations

No	Fusion partner (exons)	Mutations
1	*H3-3A* (exon 2) and *AFF2* (exon 8)	
2	*EWSR1* (exon 14) and *AFF2* (exon 8)	
3	*CHD4* (exon 10) and *AFF2* (exon 6)	
4	*NUCKS1* (exon 3) and *AFF2* (exon 8)	
5	*CHD4* (exon 10) and *AFF2 (*exon 9)	*HRAS*: c.38G > T p.(Gly13Val) 36% VAF

### Presentation, treatment, and follow-up

Case 1 (*H3-3A*::*AFF2*): The patient presented with a 2-month history of left-sided nasal obstruction and intermittent bloody rhinorrhea, reporting a mild reduction in olfactory function. Imaging revealed a tumor in the maxillary sinus extending into the middle meatus. The lesion was resected via an endonasal endoscopic approach, followed by postoperative radiotherapy (66 Gy in 32 fractions). At the 12-month follow-up, there was no evidence of disease.

Case 2 (*EWSR1*::*AFF2*): This patient had a 3-year history of bifrontal and bifacial headaches (frontal and maxillary sinus distribution), accompanied by rhinorrhea, with a notable worsening over the preceding 10 months. Two weeks before evaluation, the patient developed blurred vision and reduced color perception in the right eye, accompanied by orbital pain exacerbated by eye movement, nausea, and vomiting. This culminated in complete vision loss (no light perception) in the right eye. Over the subsequent 2 days, similar symptoms appeared in the left eye. Additionally, the patient reported an 80-pound unintentional weight loss. Imaging revealed a tumor in the right nasal cavity, extending through the cribriform plate to involve both frontal lobes and into the right orbit. Treatment consisted of radiotherapy (70 Gy in 33 fractions). At the 17-month follow-up, there was no evidence of disease.

Case 3 (*CHD4*::*AFF2*): Part of this case was previously reported by Miller et al. [6]; the current description includes newly obtained follow-up data and identification of the primary tumor site. This patient initially presented with chronic sinus infections and a nodal mass in the submandibular gland region. The tumor was diagnosed as a neck metastasis from an unknown primary and was treated with a left-sided level I–IV neck dissection, followed by adjuvant chemoradiation therapy, as previously described.

After the initial publication, additional follow-up information became available. Three years (37 months) later, the patient noticed a new swelling over the right clavicular region. In addition to the supraclavicular lesion, a CT scan revealed an enlarging cystic lesion within the left nasolacrimal duct, along with stable post-treatment changes. Excisional biopsy of a right supraclavicular lymph node confirmed a metastatic high-grade neuroendocrine tumor without extranodal extension. A biopsy of the left nasolacrimal duct also showed fragments of a high-grade neuroendocrine tumor. An FDG PET/CT scan identified a hypermetabolic lesion in the left nasolacrimal duct with ipsilateral orbital and sinonasal extension, indeterminate multiple right-sided cervical lymph nodes, and no distant disease. The patient underwent a left maxillectomy, orbital exenteration, right neck dissection, and an anterolateral thigh free flap reconstruction. Pathologic examination revealed a high-grade neuroendocrine tumor (Ki-67 approximately 30%) involving the maxilla, orbital floor, left ethmoid bone, left nasal floor soft tissue, and 8 of 35 right neck lymph nodes. Postoperatively, the patient received radiation therapy and is currently undergoing chemotherapy. Follow-up after this last treatment was not available.

Case 4 (*NUCKS1*::*AFF2*): For this case, detailed clinical information regarding presentation, radiology, treatment, and follow-up was not available due to the consultation setting in which the specimen was evaluated.

Case 5 (*CHD4*::*AFF2*): This patient presented with a 6-month history of a parotid mass, which, over time, developed tenderness to palpation. There was no prior medical history of note. Imaging revealed an irregular mass involving the superficial parotid gland, which was resected with facial nerve preservation. Pathologic examination showed a 4.2 cm parotid tumor with features of both squamoid and neuroendocrine differentiation, with the involvement of a peri-parotid lymph node. The patient is due to commence adjuvant radiotherapy.

### Morphology and immunohistochemistry

**Fig. 1:**
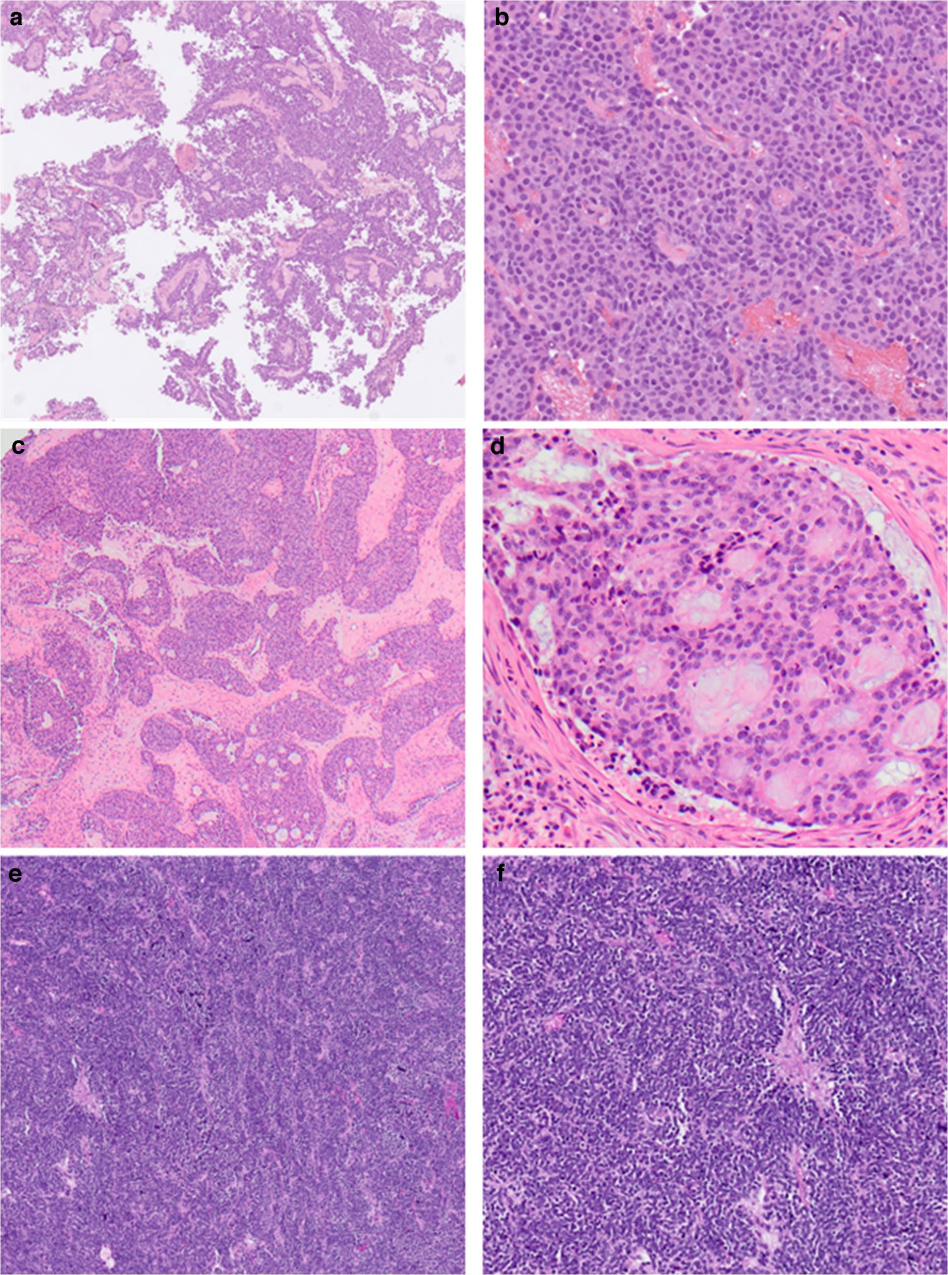
Morphological spectrum of AFF2 carcinomas. **a**, **b** Case 1 (*H3-3A*::*AFF2* fusion), a sinonasal tumor that shows bland transitional cell-like morphology with prominent acantholytic changes. **c**, **d** Case 2 (*EWSR1*::*AFF2* fusion), a sinonasal tumor with adenocarcinoma morphology. **e**, **f** Case 3 (*CHD4*::*AFF2*), primarily presented as lymph node metastasis; during follow-up, a primary in the sinonasal tract was identified; the tumor shows small blue round cell morphology

Case 1, the *H3-3A*::*AFF2* tumor showed bland monomorphous tumor cells in anastomosing sheets with squamoid morphology, moderate cytoplasm, and round nuclei (see Fig. 1a and b). There was dissociative growth at the periphery but no keratinization or glandular differentiation. Keratin AE1/3 displayed diffuse strong expression. The p40 and p63 stains were diffusely strongly positive. Keratin 5 showed variable positivity, particularly in the peripheral areas of the tumor fields. The p53 staining showed wild-type expression, and P16 was negative. The BRG1 and INI1 stains showed preserved nuclear expression. EBV in situ hybridization (EBER ISH), CD99, SOX10, synaptophysin, desmin, and NUT were negative. The AFF2 stain was positive in the tumor cells.

Case 2, the *EWSR1*::*AFF2* tumor displayed features consistent with a high-grade carcinoma, exhibiting solid, trabecular, cribriform, and glandular growth patterns (see Fig. 1c and d). Within the glandular areas, mucin production was present, but no goblet cells were present, and comedonecrosis was common. The non-glandular regions showed small round blue cell morphology with a high nuclear-to-cytoplasmic ratio. In glandular areas, the cells had a lower nuclear-to-cytoplasmic ratio, granular, lightly eosinophilic cytoplasm, and indistinct cell membranes. Across the tumor, there were numerous intratumoral neutrophils, brisk mitotic activity, and apoptotic cells. Immunohistochemistry demonstrated positivity for pooled keratin, LMWK, and focal synaptophysin and chromogranin A staining. The tumor was negative for p63, CK5/6, CD99, calretinin, CDX2, OCT4, SATB2, p16 (patchy), S100, Sox10, SMMS, SMA, NUT, desmin, MyoD, PAX8, TTF1, napsin A, PSA, and PSAP. INI1, BRG1, and ARID1A were all retained, and EBER ISH was negative. The AFF2 stain was positive in the tumor cells.

Case 3, a *CHD4*::*AFF2* tumor detected in a lymph node, consisted of neoplastic cells growing in sheets, displaying the morphology of a small round blue cell tumor characterized by scant to absent cytoplasm and round to oval nuclei with irregular borders (see Fig. 1e and f). The chromatin was coarsely clumped with variably prominent chromocenters, and there was no evidence of a specific differentiating architectural pattern. Immunohistochemistry demonstrated positive staining for synaptophysin, chromogranin, CD56, and BCL2; weak positivity for p63, SOX-11, and c-MYC; and focal positivity for CD99/O13 (mostly paranuclear, dot-like, with minimal membranous staining). The tumor was negative for pankeratin, Cam5.2, HMWCK (34-beta), S-100 protein, CK5/6, CK7, CK20, CD45, cyclin D1, CD3, CD10, CD20, CD30, CD23, CD5, MUM-1, BCL6, TTF-1, p16, p40, WT-1, desmin, beta-catenin, calretinin, and NKX2.2. SMARCB1 and SMARC4 showed intact/normal expression, while in situ hybridization for HR-HPV RNA was negative. The Ki-67 proliferation index ranged from 30 to 40%. The AFF2 stain was positive in the tumor cells.

**Fig. 2:**
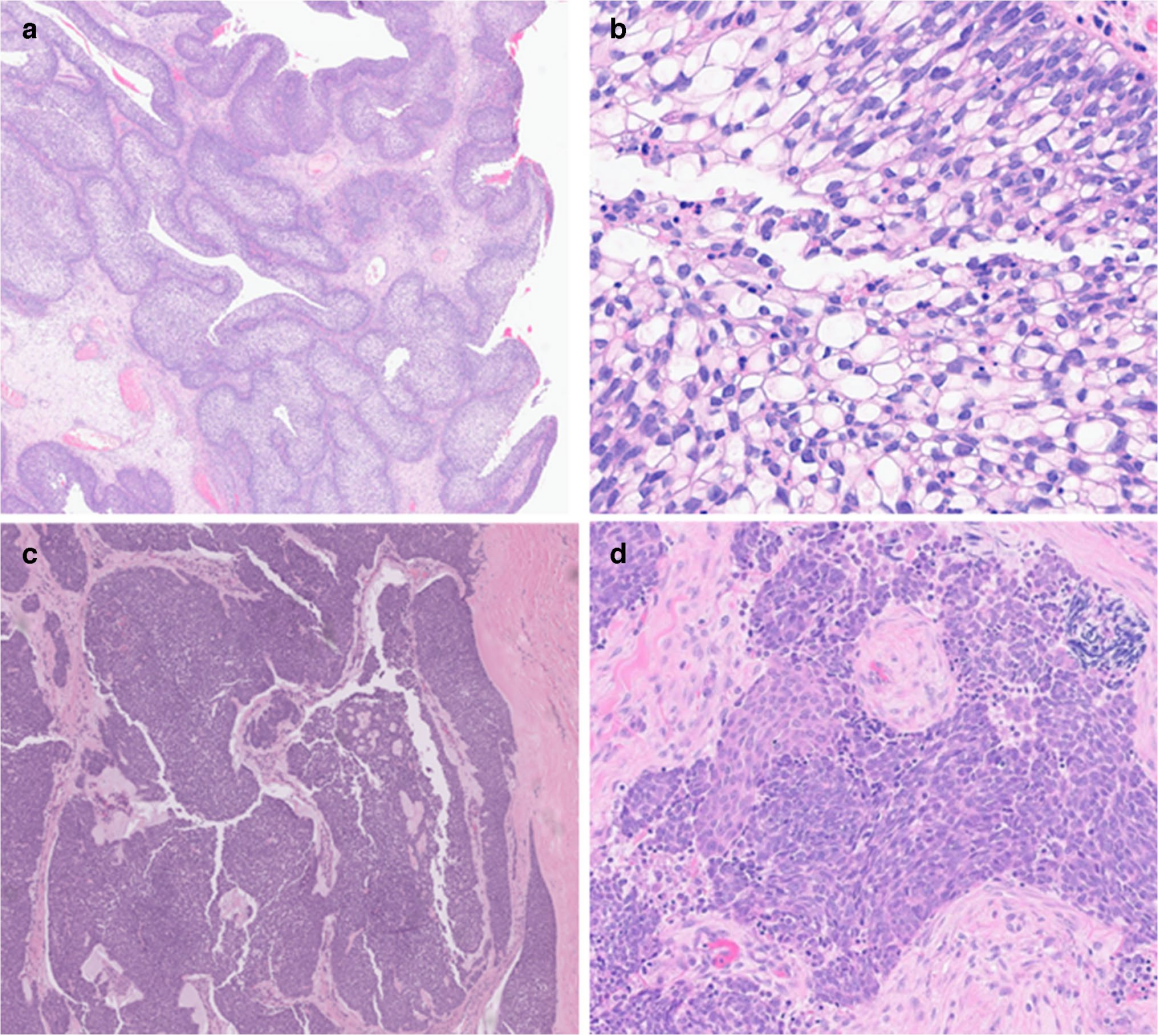
Morphological spectrum of AFF2 carcinomas. **a**, **b** Case 4 (*NUCKS1*::*AFF2*), located in the sinonasal tract, shows transitional cell-like morphology with clear cell changes. **c**, **d** Case 5 (CDH4::AFF2) located in the parotid, the tumor shows mixed squamoid and neuroendocrine differentiation

Case 4, the *NUCKS1*::*AFF2* tumor, showed architecture resembling an inverted papilloma, with some similarities to transitional cell epithelium, with brisk exocytosis of neutrophilic granulocytes (see Fig. 2a and b). However, there were extensive clear cell changes without evidence of pleomorphism or other high-grade features. EBER ISH and p16 were negative. The AFF2 stain was positive in the tumor cells.

Case 5, the *CHD4*::*AFF2* tumor in the parotid, comprised jagged islands of basaloid cells with appearances suggestive of a non-keratinizing squamous cell carcinoma (see Fig. 2c and d). Features of neuroendocrine change, including increased proliferation (mitotic rate > 10 mitoses/mm^2^) with speckled nuclear chromatin and reduced nuclear to cytoplasmic ratio, were seen throughout. Focal squamous morules were identified. The tumor involved a periparotid lymph node and showed lymphovascular and perineural invasion. Immunohistochemistry demonstrated diffuse expression of pan-cytokeratin, synaptophysin, chromogranin, p63, CK5, and p40, with a more focal expression of CAM5.2 and INSM1. CK20, CK7, TTF-1, calcitonin, EBER ISH, and HPV in situ hybridization (HPV ISH) were negative. The Ki-67 proliferation index was 10–15%. The AFF2 stain was positive in the tumor cells.

## Discussion

*DEK*::*AFF2* carcinoma presents a diagnostic challenge, mainly due to its morphological overlap with other sinonasal tumors, underscoring the essential role of molecular testing for accurate diagnosis in difficult-to-classify cases. Thus far, some homogeneity could be appreciated in the reported cases, with similarities to inverted papilloma and non-keratinizing squamous cell carcinoma being observed. We show more heterogeneous morphological, immunohistochemical, and fusion-partner patterns. Three carcinomas in this series showed focal or diffuse neuroendocrine expression; one even lacked keratin expression. These histologic and immunophenotypic patterns may intersect with those of other tumors, complicating the diagnostic process.

This further complicates diagnosing these tumors, as even identifying more conventional *DEK*::*AFF2* carcinomas can be challenging. This type of carcinoma typically demonstrates characteristic histologic features, including a mixed exophytic and endophytic growth pattern, monomorphic transitional cell-type cytomorphology, acantholytic changes, and prominent neutrophilic infiltration [18–20]. The immunohistochemical profile of more conventional cases is similar to other non-keratinizing squamous cell carcinomas, with a diffuse expression of p40, p63, cytokeratin 5/6, and cytokeratin AE1/AE3. One significant diagnostic issue involves the frequent resemblance of *DEK*::*AFF2* carcinoma to sinonasal papilloma, especially troublesome in small biopsy samples, leading to potential delays in proper diagnosis and subsequent treatment. Accurate differentiation between *DEK*::*AFF2* carcinoma and inverted papilloma is crucial, as *DEK*::*AFF2* carcinoma is associated with a more aggressive clinical course, including higher risks of local recurrence and metastasis, and survival rates similar to conventional squamous cell carcinoma [21].

Some documented *DEK*::*AFF2* carcinomas exhibit more pronounced malignant characteristics, such as increased cytologic atypia, necrosis, and an infiltrative growth pattern, which may mimic conventional non-keratinizing or basaloid squamous cell carcinoma [5, 22]. Other entities with similar histologic features should be excluded in such cases, including SWI/SNF-deficient tumors, NUT carcinoma, and adamantinoma-like Ewing sarcoma. Immunohistochemical markers, particularly pan-cytokeratin, p40, neuroendocrine markers, INI1, BRG1, NUT, and CD99, are integral to achieving accurate differentiation [23]. Three out of five cases of our series exhibit high-grade characteristics and unusual immunophenotypes, necessitating molecular analysis to suggest these tumors fall into this *AFF2*-fused carcinoma spectrum.

All tumors in this series involved *AFF2* rearrangement with fusion partners other than *DEK*, suggesting that the fusion partner may influence specific morphologies and immunophenotypes. For instance, the fusion partner *NUCKS1* might be associated with clear cell morphology, whereas *CHD4* could be linked to a small round blue cell neuroendocrine phenotype. However, a recently reported *DEK*::*AFF2* carcinoma also exhibited undifferentiated morphology and neuroendocrine differentiation [24], suggesting that this relationship may not be strictly partner-dependent. Larger series will be necessary to determine whether specific fusion partners define consistent phenotypic subsets or whether a broader, overlapping morphologic spectrum exists.

If *DEK*::*AFF2* carcinoma is suspected, the fusion can be proven by cytogenetic or molecular analysis, such as fluorescence in situ hybridization or RNA generation. However, specific and sensitive immunohistochemistry targeting the AFF2 C-terminus has been described [20, 22]. All five cases in this series showed positive AFF2 staining, suggesting utility as a screen for all AFF2 carcinomas regardless of partner gene, similar to the use of NUT immunohistochemistry for NUT carcinoma. Time will tell if its specificity is sufficiently optimal, as a recent study also described weak staining patterns in some tumors without the fusion [25].

As the molecular landscape of *DEK*::*AFF2* carcinoma becomes increasingly elucidated, recognizing its diverse morphological and clinical profiles is critical. While these tumors are predominantly reported in the sinonasal, temporal bone, and skull base head and neck regions, our series includes one tumor located in the parotid gland, in addition to one reported *AFF2*-rearranged carcinoma in the thoracic region; this suggests a broader anatomical distribution, similar to NUT carcinoma, which is known to present across multiple organ systems [26, 27]. Currently, the diagnosis of *DEK*::*AFF2* carcinoma can be suggested based on its characteristic histologic appearance, particularly in sinonasal cases with classic features. However, as non-canonical cases emerge and as new fusion partners are identified, molecular confirmation may be necessary in selected settings, with AFF2 immunohistochemistry potentially serving as a valuable adjunct.

Given the consistent involvement of *AFF2* as a fusion partner and the distinct immunohistochemical expression of AFF2 in these tumors, we propose the term “*AFF2* carcinoma” to describe this entity. However, the presence of neuroendocrine differentiation in some cases—often associated with more aggressive behavior in other head and neck contexts—raises the question of whether these tumors should be unified within a single category or considered as separate entities.

## Data Availability

Not applicable.
